# Spontaneous rupture of a renal artery pseudoaneurysm in a hemodialysis patient: A case report

**DOI:** 10.1097/MD.0000000000025970

**Published:** 2021-05-21

**Authors:** Seunghye Lee, Sehyun Jung, Hyun-Jung Kim, Ha Nee Jang, Dong Jun Park, Eunjin Bae, Tae Won Lee, Se-Ho Chang

**Affiliations:** a Department of Internal Medicine-Nephrology, College of Medicine, Gyeongsang National University; b Department of Internal Medicine-Nephrology, Gyeongsang National University Hospital; c Institute of Health Sciences, Gyeongsang National University, Jinju; d Department of Internal Medicine-Nephrology, Gyeongsang National University Changwon Hospital, Changwon, Republic of Korea.

**Keywords:** case report, hemodialysis, percutaneous embolization, renal artery pseudoaneurysm, spontaneous rupture

## Abstract

**Rationale:**

Renal artery pseudoaneurysm is a rare vascular lesion usually caused by trauma or percutaneous urological procedures. Spontaneous rupture of pseudoaneurysms without predisposing events, especially in hemodialysis patients, has rarely been reported.

**Patient concerns:**

A 25-year-old man receiving maintenance hemodialysis visited the emergency room because of sudden severe right flank pain. He had no history of trauma or urological procedures except for a left renal biopsy to diagnose Alport syndrome 10 years prior.

**Diagnosis:**

Contrast-enhanced computed tomography revealed a right perirenal hematoma with pseudoaneurysms.

**Interventions:**

On renal angiography, multiple pseudoaneurysms were observed in the right renal artery branches and embolization was performed.

**Outcomes:**

Post-angiography showed no pseudoaneurysms. His abdominal pain improved, and he was discharged 2 weeks after embolization.

**Lessons:**

When maintenance dialysis patients complain of severe abdominal pain, spontaneous rupture of a renal pseudoaneurysm should be considered as a differential diagnosis, even if the patient has no history of trauma or previous urological procedures.

## 1 Introduction

Spontaneous retroperitoneal hemorrhage is a rare, but potentially fatal condition in patients undergoing hemodialysis.^[1]^ Most spontaneous retroperitoneal hemorrhages are kidney origin. Renal bleeding usually extends into the renal collecting system, leading to hematuria, or into the perinephric space, leading to perirenal hematoma, or retroperitoneal hemorrhage.^[2]^ Renal artery pseudoaneurysm (RAP) is a rare cause of renal bleeding. In particular, spontaneous rupture of pseudoaneurysms without predisposing events in hemodialysis patients has rarely been reported. We report a case of spontaneous rupture of RAP treated with embolization in a patient with Alport syndrome undergoing hemodialysis.

## 2 Case report

**Figure 1:**
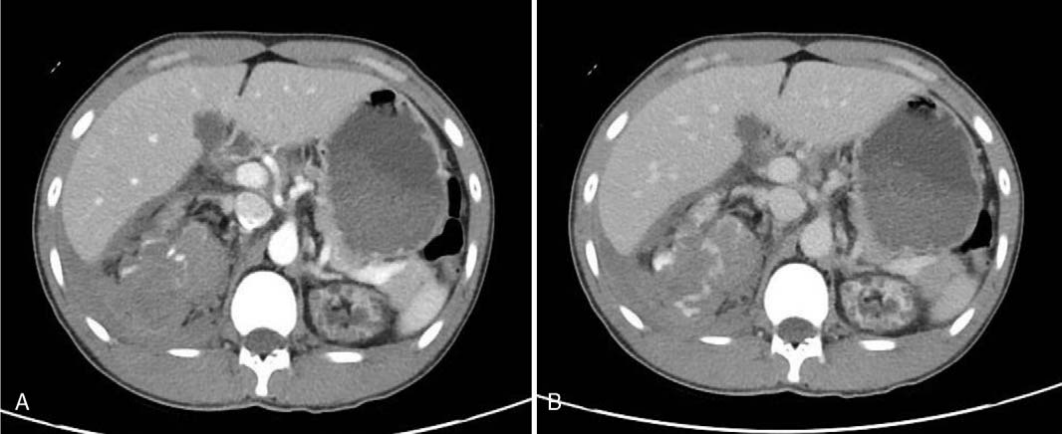
Contrast-enhanced CT showing a right perirenal hematoma with pseudoaneurysm, retrohemoperitoneum, and hemoperitoneum. (A) Portal phase. (B) More contrast dye leakage was observed on delayed-phase imaging.

**Figure 2:**
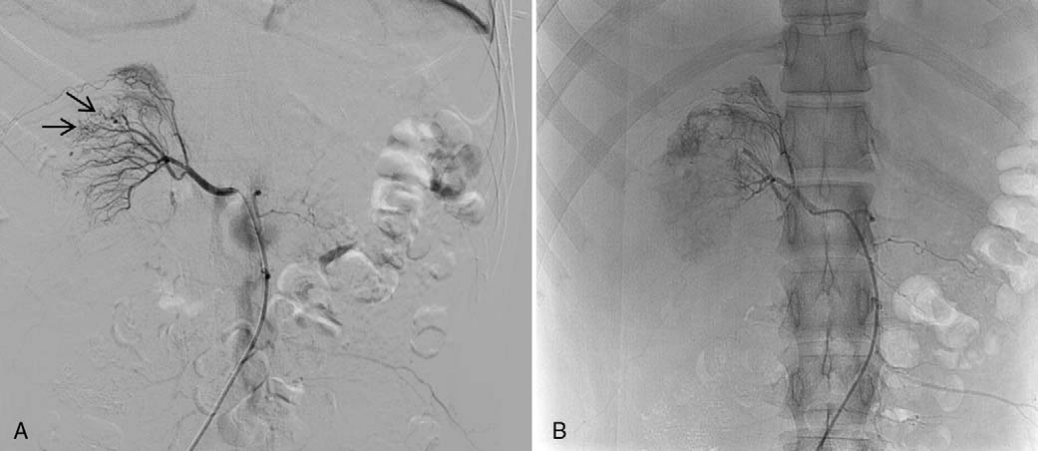
Renal angiography images. (A) Multiple pseudoaneurysms (black arrow) were observed in the right renal artery branches. (B) Embolization was performed, and post-angiography imaging showed no pseudoaneurysms or contrast leakage.

A 25-year-old man visited the emergency room because of sudden severe right flank pain. He was diagnosed with end-stage renal disease due to Alport syndrome and had been receiving maintenance hemodialysis 3 times a week for 4 hours per session since 2013. The last hemodialysis session was the previous day, and 1000 units of heparin were administered as an initial bolus dose followed by an infusion of 500 units per hour of heparin 3 times as a maintenance dose. He had no history of trauma or urological procedures except for a left renal biopsy to diagnose Alport syndrome 10 years prior. His blood pressure was well controlled by taking antihypertensive drugs and undergoing regular hemodialysis. The patient had no other symptoms or signs during hemodialysis. His vital signs at the emergency room were as follows: blood pressure, 160/100 mm Hg; heart rate, 77 beats per minute; body temperature, 36.5°C; and respiratory rate, 20 breaths per minute. Initial laboratory findings revealed white blood cell count 9080/mm^3^, hemoglobin 11.7 g/dL, hematocrit 35%, platelets 203,000/μL, blood urea nitrogen 26 mg/dL, creatinine 9.32 mg/dL, erythrocyte sedimentation rate 5 mm/hour, and C-reactive protein 0.9 mg/L. His prothrombin time and activated partial thromboplastin time were within the normal ranges. He complained of progressively worsening right flank pain, and his hemoglobin level also decreased to 10.1 g/dL. Contrast-enhanced computed tomography revealed a right perirenal hematoma with pseudoaneurysms (Fig. 1). On renal angiography, multiple pseudoaneurysms were observed in the right renal artery branches, and embolization was performed (Fig. 2). Post-embolization angiography revealed no pseudoaneurysms or contrast leakage.

**Figure 3:**
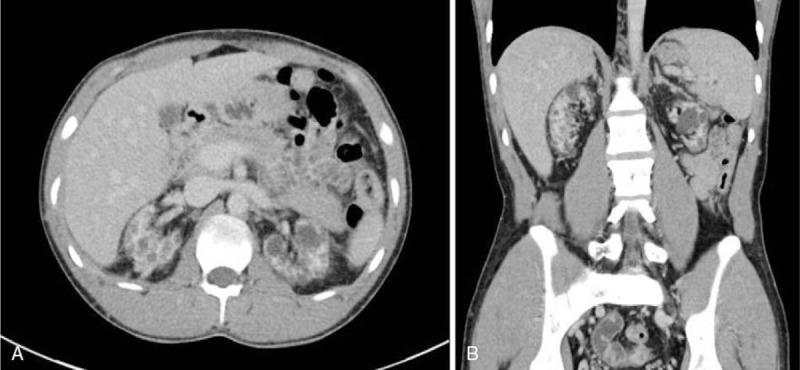
Three-month follow-up CT scan showing complete resolution of right perirenal hematoma. (A) Axial view. (B) Coronal view.

The size of the perirenal hematoma slightly decreased on CT 10 days after embolization. He had no more abdominal pain, and his hemoglobin level did not change. The patient was discharged with instructions to maintain heparin-free hemodialysis. On outpatient blood tests 3 weeks later, his hemoglobin level was 11.6 g/dL, and he did not report any discomfort. After 3 months, we performed another CT scan, which confirmed that the perirenal hematoma and retrohemoperitoneum had completely resolved (Fig. 3). The patient started using heparin again at the previous dose during hemodialysis. He had no recurrence of symptoms even after heparin was resumed during hemodialysis.

## 3 Discussion

A pseudoaneurysm is the dilatation of an artery with disruption of one or more layers of the vascular wall.^[3]^ It communicates to the intravascular space, and the external boundary of the extravascular hematoma is composed of attached extravascular tissue.^[4]^ RAP is a localized dilation of the renal artery and/or branches. RAPs can either be congenital or acquired. Congenital RAPs have been associated with autosomal dominant polycystic disease, fibromuscular dysplasia, and tuberous sclerosis.^[5]^ Acquired RAPs have been reported as a result of blunt trauma,^[6]^ penetrating trauma, and urological interventions such as partial nephrectomy,^[7]^ percutaneous nephrolithotomy,^[8]^ renal biopsy,^[9]^ and renal transplantation.^[10]^ Malignancy, coagulopathy, radiation, cyclophosphamide use,^[11]^ uncontrolled hypertension,^[12]^ and systemic vasculitis such as Behcet's disease^[13]^ have also been reported as contributing factors. In previously reported cases, the longest interval between initial trauma and the diagnosis of aneurysm rupture was 15 years.^[14]^ In the present case, the patient had no history of trauma for at least 15 years, except for a left kidney biopsy 10 years prior. RAP occurred in the right kidney, and it was not associated with a left kidney biopsy. Blood pressure was well controlled, and there was no evidence of malignancy, coagulopathy, or systemic vasculitis.

One previous report suggested that end-stage renal disease patients may develop platelet dysfunction, which increases the risk of pseudoaneurysm formation after any type of arterial injury.^[15]^ In addition, anticoagulants have been suggested as risk factors for pseudoaneurysms in several reports.^[16,17]^ One report of spontaneous splenic rupture in a hemodialysis patient also suggested that heparin use was an important risk factor for splenic rupture.^[18]^ We could not find any history of predisposing events that could cause renal artery injury in the patient. He did not take any anticoagulant drugs. However, he was a patient with end-stage renal disease and had been using heparin for a long period of time during hemodialysis. These 2 factors may precipitate pseudoaneurysms.

Pseudoaneurysms are difficult to diagnose because of the lack of specific clinical signs or symptoms. However, early detection and treatment are important, as rupture of pseudoaneurysms can cause life-threatening hemorrhages.^[19,20]^

Indications for treatment include hemorrhage, uncontrolled hypertension, progressively worsening pain, enlargement of the pseudoaneurysm, and presence of an arteriovenous fistula.^[21]^ Currently, endovascular treatment including embolization (as chosen in this case) is the intervention of choice in most emergency circumstances.^[22]^ There are no established protocols for post-treatment follow-up of patients who have undergone selective angioembolization.^[20]^

In patients undergoing hemodialysis, it is important to determine when heparin is reused during hemodialysis. The timing and necessity of CT follow-up have not been discussed previously. We confirmed complete disappearance of the hematoma on CT after 3 months of treatment, and decided to start using heparin. If hematoma still remains in the abdominal cavity, heparin should be used carefully during hemodialysis, considering the risk of re-bleeding.

## 4 Conclusion

RAPs are rare in patients undergoing hemodialysis. When maintenance dialysis patients complain of severe abdominal pain, spontaneous rupture of a renal pseudoaneurysm should be considered as a differential diagnosis, even if the patient has no history of trauma or previous urological procedures.

This is an unusual case of spontaneous renal pseudoaneurysm rupture in a hemodialysis patient without a predisposing event that was successfully managed with embolization.

## Author contributions

**Conceptualization:** Seunghye Lee, Se-Ho Chang.

**Supervision:** Sehyun Jung, Dong Jun Park, Eunjin Bae, Tae Won Lee.

**Writing – original draft:** Seunghye Lee, Ha Nee Jang.

**Writing – review & editing:** Hyun-Jung Kim, Se-Ho Chang.
